# A dual-instrument Kalman-based tracker to enhance robustness of microsurgical tools tracking

**DOI:** 10.1007/s11548-024-03246-4

**Published:** 2024-08-12

**Authors:** Mattia Magro, Nicola Covallero, Elena Gambaro, Emanuele Ruffaldi, Elena De Momi

**Affiliations:** 1 Department of Electronics, Information and Bioengineering, Politecnico di Milano, Milan, Italy; 2 Medical Microinstruments, Inc., Wilmington, USA

**Keywords:** Deep learning, Detection, Kalman filter, Tracking, Microsurgery

## Abstract

****Purpose**:**

The integration of a surgical robotic instrument tracking module within optical microscopes holds the potential to advance microsurgery practices, as it facilitates automated camera movements, thereby augmenting the surgeon’s capability in executing surgical procedures.

****Methods**:**

In the present work, an innovative detection backbone based on spatial attention module is implemented to enhance the detection accuracy of small objects within the image. Additionally, we have introduced a robust data association technique, capable to re-track surgical instrument, mainly based on the knowledge of the dual-instrument robotics system, Intersection over Union metric and Kalman filter.

****Results**:**

The effectiveness of this pipeline was evaluated through testing on a dataset comprising ten manually annotated videos of anastomosis procedures involving either animal or phantom vessels, exploiting the Symani®Surgical System—a dedicated robotic platform designed for microsurgery. The multiple object tracking precision (MOTP) and the multiple object tracking accuracy (MOTA) are used to evaluate the performance of the proposed approach, and a new metric is computed to demonstrate the efficacy in stabilizing the tracking result along the video frames. An average MOTP of 74±0.06% and a MOTA of 99±0.03% over the test videos were found.

****Conclusion**:**

These results confirm the potential of the proposed approach in enhancing precision and reliability in microsurgical instrument tracking. Thus, the integration of attention mechanisms and a tailored data association module could be a solid base for automatizing the motion of optical microscopes.

## Introduction

Microsurgery is a surgical field that focuses on performing high-demand procedures on small-scale structures, typically involving blood vessels, nerves, and other delicate tissues, under suitable optical microscope guidance [1]. The success of microsurgical interventions relies on the surgeon’s ability to manipulate specialized tools with precision and dexterity. Thus, the introduction of robotic systems permits to get an improved surgical precision by tremor filtration and motion scaling, and an enhanced manipulation and flexibility for the surgeon [2, 3].

**Fig. 1:**
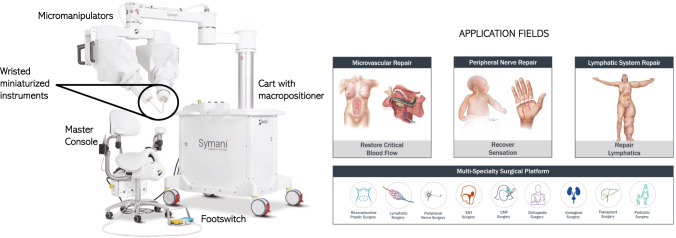
Symani®Surgical System. On the left, an overview of the structure and the main components is showed. On the right, the general application fields and the specific ones are depicted

An example is the Symani®Surgical System (Fig.  1, left), developed by Medical Microinstruments, Inc., [Wilmington, USA], which is a robotic and ergonomic platform designed for microsurgery as free flap reconstructions, lymphatic surgery, nerve reconstruction or re-plantation (Fig. 1, right). The surgeon operates from a remote console consisting of a chair, a foot switch and two joysticks, through which occurs the manipulation of the two robotic arms. This system aims to elevate the standard of microsurgical procedures, improving the surgeon’s natural dexterity and range of motion beyond the capability of the human hand, thanks to the world’s smallest and wristed surgical microinstruments [4].

The anatomical scene is displayed to the surgeon by an optical system which should be manually controlled. This may result in limited accuracy and instability of motion, and fatigue for the surgeon that leads to a decline in hand–eye coordination and precision. Furthermore, manual control may not provide optimal ergonomic conditions, leading to discomfort and potentially impacting performance over the course of a lengthy procedure. Thus, the implementation of automatic camera control represents a significant advancement within the existing framework of microsurgery practice [5].

To leverage this approach, it is essential the development of automated systems, capable of precisely executing the detection, recognition, and tracking of surgical instruments within a small, cluttered, and complex environment typical of microsurgery [6]. Thus, an instrument tracking module, embedded in optical camera, can enable dynamic synchronization between instruments motion and optical microscope’s orientation. This outcome is essential to ensure that the camera’s field of view accurately follows the surgical action, permitting optimal visualization during the procedure [7]. This can improve the accuracy of the location of surgical tools, enhance the control capabilities of the surgeons performing the procedures, minimizing the risk of damage to delicate tissues, and reduce the cost of human assistants; collectively, these advantages make surgery more efficient [8].

In this work, we propose a dual-instrument tracker for the two robotic arms of the Symani®Surgical System, also applicable to other instruments of a similar nature. This tracker is composed of a novel detection module based on SAM that can provide a real-time analysis of visual information, minimizing the quantity of undetected instruments. In cascade, a robust and tailored data association module to recognize an instrument as identical along the frames is inserted.

This paper is organized as follows: Sect. 2 provides a short review of literature in the research area of tracking. Section 3 describes the proposed tracking approach, while Sect. 4 presents the experimental protocol and the exploited metrics for validation. The efficacy of the described method is demonstrated in Sect. 5, and, finally, Sect. 6 provides a summary of the outcomes and discusses future improvements.

## Related works

A surgical tracker, based on tracking-by-detection paradigm, is divided in two main steps: instances detection and instances association in consecutive frames. First, instances, i.e., surgical instruments, are detected in an image or video frame exploiting a detector, which localizes, using bounding boxes, and classifies each instance [9]. Then, each instance is matched in the consecutive frames based on similarity scores, also employing modules to predict the locations of instruments in case of detection failure [10]. As a result, an instrument tracking module that focuses on continuously pursuing each instance over time is obtained.

### Detection module

The advent of deep learning revolutionized surgical instrument detection by enabling the development of sophisticated neural network architectures [11–13] overcomes the performance of traditional methods, in cases of complex environments or objects exhibiting diverse appearances [14]. Convolutional neural networks (CNNs) have been extensively utilized for their ability to automatically learn hierarchical features from raw pixel data, leading to improved detection accuracy [15]. In the literature, two-stage and one-stage CNN detectors can be found: the latter detects all the instances of interest at once, while the former requires an additional region proposal-based network (RPN) to select a specific region of the image in which performing detection.

Sarikaya et al. [16] proposed a RPN and a multimodal two-stream convolutional network for surgical instrument detection, combining image and temporal motion cues jointly. In [17], to detect laparoscopic surgical instruments, a modular anchor network based on faster R-CNN is developed. In [18], the frame-by-frame real-time surgical instrument detection has been developed by cascading two different CNN networks: an hourglass network that outputs detection heatmaps for tool-tip area representation and a modified visual geometry group (VGG) network for creating a bounding box around the detected part. These two networks jointly predict the localization.

While the accuracy of two-stage CNN algorithms is higher, most of the algorithms’ real-time performance is still slightly worse than the one of one-stage techniques. In [19], a one-stage CNN algorithm based on regression has been developed to achieve surgical instrument recognition and tracking. By downsampling, the CNN model could identify and follow surgical instruments in real time with fewer parameters. Colleoni et al. [20] proposes an encoder–decoder architecture for surgical instrument joint detection and localization, exploiting spatiotemporal features. In [21], it has been proposed a one-stage instrument detection framework controlled by reinforcement learning. Shi et al. [22] develops a CNN enhanced with real-time attention guidance for frame-by-frame detection of surgical instruments in surgical videos. In their paper, Beal et al. [23] addressed the use of the transformer architecture, i.e., a neural network model based on the self-attention mechanism, for the detection tasks. Liu et al. [24] proposed FENet, an enhanced feature-fusion network for real-time surgical tool detection.YOLOv5 (You Only Look Once) neural network is a state-of-the-art object detection model, renowned for its efficiency and accuracy in real-time applications [25]. Developed by Ultralytics [Los Angeles, CA, USA], YOLOv5 introduces an architecture based on CSP-Darknet53 and PANet that lead to notable improvements in speed and precision. This deep learning model employs a single neural network to simultaneously predict bounding boxes and classify objects within them, eliminating the need for multiple stages [26]. YOLO-NAS [27] was released in May 2023 by Deci, a company that develops production-grade models and tools to build, optimize, and deploy deep learning models. This network is designed to detect small objects, improve localization accuracy, and enhance the performances thanks to the application of neural architecture search (NAS) to refine the architecture of an already trained model and the quantization aware blocks.

### Data association module

One of the simplest tracking-by-detection methods is simple online and real-time tracking (SORT), which employs solely a Kalman filter as a motion model, the Hungarian algorithm as association method and the IoU as similarity metric [28]. A more advanced version is DeepSORT, which improves the association component by introducing the cosine distance of appearance features as matching cost [29]. To accomplish quick 2D tracking of surgical instruments, Qiu et al. [30] applied a multi-domain convolutional neural network and a focus loss to reduce the effect of clearly identifiable negative instances. Using a CNN with a line segment detector (LSD) and spatiotemporal context (STC) learning, Chen et al. [31] presented a visual tracking technique for two-dimensional tool detection. All these networks initialize the tracker only once, and then the features of the instruments are tracked in each frame, avoiding the detection frame-by-frame. While this methodology yields rapid algorithms, it introduces inconsistencies in detection and renders it impractical to re-track an instrument that temporarily exits the field of view only as the same instance.

The novelty of the proposed approach is the usage of a detection module which aims at minimizing the inaccuracy provoked by motion blur, adverse lighting conditions, specular reflections on the tool surfaces and on the tissue regions, and shadows cast by the tool. Furthermore, a robust data association module that leverage Kalman filter and image-based constrains is developed to achieve an equilibrium between tracking precision and real-time responsiveness. Furthermore, the introduction of a re-tracking, designed to monitor the robotic arm’s motion, is essential in this work to enable consistent identifier association.

## Material and methods

**Fig. 2:**
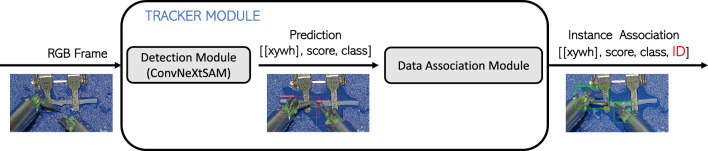
Overview of the proposed pipeline: the tracker module takes as input RGB video frame. Then, a detection module, after suitable preprocessing, performs prediction of the position of maximum two robotics arms of the Symani®Surgical System. Finally, at each of the arms detected, the data association module adds an identifier that remains coherent across consecutive frames

Section 2 defines a tracker module as a combination of detection module and data association module. In the proposed implementation (Fig. 2), these two modules are run in sequence on each RGB frame of a video, providing as output detection and identification of each instruments. In a nutshell, the objective is to detect a maximum of two robotic arms in the frame, exploiting a deep network for the detection, while ensuring that they satisfy certain criteria: they must not enter the field of view from the upper boundary to ensure symmetry with the surgeon’s manipulation tools, and they must not intersect in the first frame.

### Detection module

**Fig. 3:**
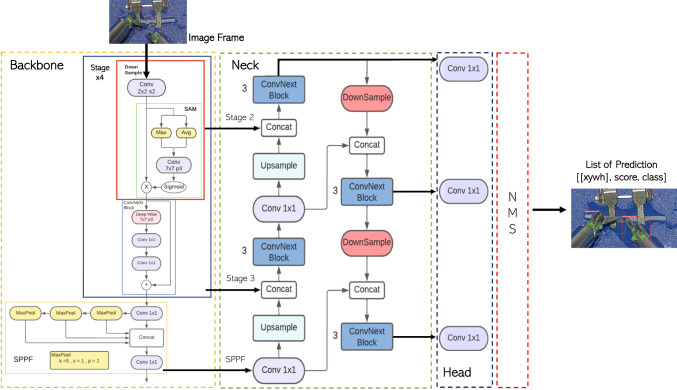
The proposed architecture for the detection module is constituted of four repetition of the blocks DownSample, spatial attention module and ConvNeXt, with the aim of extracting features at different levels. The final block is a spatial pixel pair features module (SPPF) that creates pixel pairs. Three outputs at different levels of this main structure are conveyed to the neck structure, which is composed of a feature pyramid network coupled with a path aggregation network. As output, activation maps are provided to the three heads of the network, which, thus, provided the final prediction of the bounding boxes. These predictions are filtered by a non-maximum suppression (NMS) module to maintain only the valuable ones

ConvNeXtSAM represents the proposed methodology for detecting surgical instruments and it is embedded in the detection module of Fig. 2. We modify the YOLOv5 state-of-the-art architecture, aiming to reduce the occurrence of missed detection and imprecise bounding box predictions, while maintaining a comparable level of detection accuracy. Furthermore, the objective is to enhance precision in the accurate localization of elongated instruments that are in close proximity, a scenario commonly encountered in anastomosis procedures. Thus, we introduced a combination of ConvNeXt and spatial attention module (SAM), followed by a Path Aggregation Feature Pyramid Network (FPN–PAN), trying to mimic the Transformer approach. Then, activation maps at three different levels are provided to the heads of the network to perform instances regression. A non-maximum suppression (NMS) module is inserted to maintain only the valuable instances. The components of the network are detailed in Fig. 3.

### Data association module

The aforementioned network generates a set of predicted instances, namely bounding boxes for each robotic surgical instrument. These predictions are then fed to the data association module, which is implemented leveraging Kalman filter and image-based constrains. The primary objective of this module is to allocate a unique identifier (ID) to each input instrument, and this ID is associated to the same instance in subsequent frames.

**Fig. 4:**
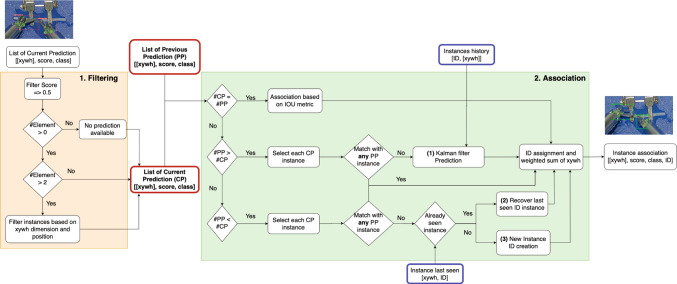
Flowchart of data association module divided in two main steps: filtering and association. The first step receives as input the prediction of the instances’ locations from the detection module and allows to have, as output, a list of maximum two predictions for each frame (CP). The association tries to find a match between instances of consecutive frames

As depicted in Fig. 4, the key operations of the data association module involve filtering out weak detection and establishing associations.

**Fig. 5:**
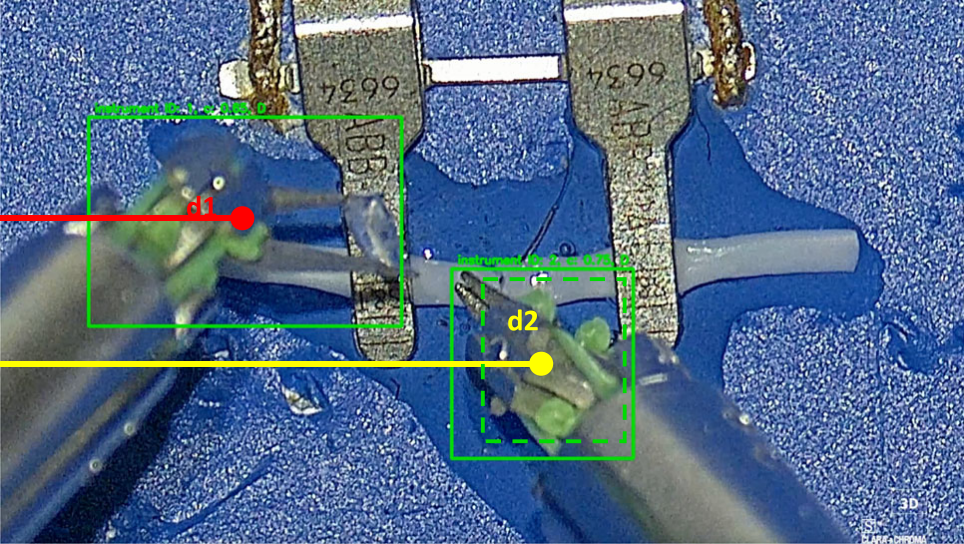
This picture shows an example of discarded instance (green dashed box), given its position. Furthermore, it is highlighted the computation of the distance of each detected instance from the image’s left edge: ID 1 is associated to the instance with lower distance (min((d1.d2)) or if we have only one detected instance, while ID 2 is assigned to the instance with greater distance (max((d1.d2))

The initial component of this process involves discarding predictions with a score (or confidence) level below a threshold of 0.5. This step reinforces the output of the NMS carried out at the conclusion of the detection module (see Fig. 3). Then, the number of detection is clipped to the maximum allowed number of instances per frame, i.e., the number of the robotic arms of the system, and this operation is based on the relative position between the instances (Fig. 5) and on the dimension of each instance with respect to the image dimension.

To each detected instrument is then assigned a distinctive ID value, ranging from 1 to 2. Specifically, ID 1 should be associated to left arm and ID 2 to right arm. This association is established in the initial frame by assessing the relative position of the instances and their distances from the image’s left edge (Fig. 5). In formula:

1 $$\begin{aligned} ID = {\left\{ \begin{array}{ll} 1, &{} \text {if } \textrm{unique} \hspace{1ex}\text {v}\hspace{1ex}d1 < d2 \\ 2, &{} \text {if } d1 > d2 \end{array}\right. } \end{aligned}$$

where *d*1 and *d*2 are the distance of the instances respect to the frame’s left edge (see Fig. 5). This is based on the assumption that the two arms should not appear crossed in the first frame of the video, as stipulated in the introductory of Sect. 3. In case where only one instrument is present, ID 1 is associated, and when a new instrument is detected, this assignment is checked and eventually corrected to be consistent with the defined classification. For subsequent frames, a comparison between current prediction (CP) and previous prediction (PP) is performed (Fig. 4), and in case where an equal number of predictions arise, the optimal match with a prior detection is sought for each current detection, based on the IoU metric. Consequently, the same ID is assigned to the instrument with highest IoU, and the resultant instance constitutes a weighted sum of the two consecutive matched instances:

2 $$\begin{aligned} \textrm{box}_i = \alpha \cdot \textrm{box}_i{}_-{}_1 + (1-\alpha ) \cdot \textrm{box}_i \end{aligned}$$

where *i* represents the current frame, *box* denotes the xywh coordinates of both the detected instrument and the matching one, and $\alpha$ is the weight parameter, which is proportional to the IoU value. A greater overlap between instances results in a higher weight, and, thus, the transition between frames will be smoother. Conversely, lower value of overlap means a not negligible motion and so, a major contribute of the current instance.

If a mismatch between the number of current detection and the previous one is identified, the potential absence of at least one match could occur (Fig. 4). Consequently, the data association module endeavors to implement three different actions.

(1) Predict the absent instance’s value in the current frame through the motion model based on Kalman filter. This filter continuously updates its state, comparing its predicted state, based on a constant velocity model, and the real location of the instance provided by the detection module. When a detection is missing, the prediction of the Kalman filter is used and it is accepted only if bounding boxes’ motion does not converge toward the image edges and the boundaries of the bounding boxes are not touching the edges for more than two consecutive frames. Indeed, in that cases, we can assume that the instrument is outside the field of view of the camera. Then, the last real information of the instance is stored with the aim of possibly find an association in future frames. The Kalman filter is a recursive and optimal estimation algorithm widely used for tracking and state estimation. The state vector $\textbf{x}$ is defined as the coordinates (*x*, *y*, *w*, *h*) representing the position and size of the bounding box. The state transition and observation models are given by:

$$\begin{aligned} \text {State Transition Model:} \quad \textbf{x}_{k}&= \textbf{F} \textbf{x}_{k-1} + \textbf{w}_{k-1} \\ \text {Observation Model:} \quad \textbf{z}_{k}&= \textbf{H} \textbf{x}_{k} + \textbf{v}_{k} \end{aligned}$$

where

- $\textbf{x}_{k}$ is the state vector at time step *k*,
- $\textbf{F}$ is the state [4x4] transition matrix based on a constant velocity model,
- $\textbf{w}_{k-1}$ is the process noise at time step $k-1$,
- $\textbf{z}_{k}$ is the observation vector at time step *k*,
- $\textbf{H}$ is the observation matrix,
- $\textbf{v}_{k}$ is the measurement noise at time step *k*.

The state transition model predicts how the system evolves over time, while the observation model establishes the connection between the predicted state and the actual measurement, allowing the Kalman filter to iteratively refine its estimates. The Kalman filter predicts the next state $\hat{\textbf{x}}_{k|k-1}$ using the state transition model and corrects it with the actual observation $\textbf{z}_{k}$ to obtain the updated state estimate $\hat{\textbf{x}}_{k|k}$:

$$\begin{aligned}&\!\,\text {Predict:} \quad \hat{\textbf{x}}_{k|k-1} = \textbf{F} \hat{\textbf{x}}_{k-1|k-1} \\&\text {Correct:} \quad \hat{\textbf{x}}_{k|k} = \hat{\textbf{x}}_{k|k-1} + \textbf{K}_{k}(\textbf{z}_{k} - \textbf{H}\hat{\textbf{x}}_{k|k-1}) \end{aligned}$$

where $\hat{\textbf{x}}_{k-1|k-1}$ is the predicted state at time step $k-1$, $\textbf{K}_{k}$ is the Kalman gain that considers covariance matrices associated with measurements and process noises, both set to a value of 2 pixels, since we expect few pixels in localization error and a small deviation from the linear motion model.

**Fig. 6:**
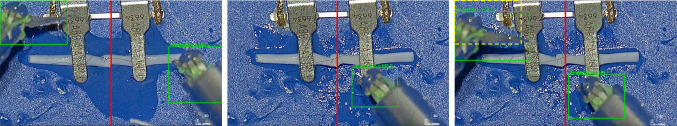
Process of ID reassignment: when an instance exits from the surgical field and then comes back, if the Intersection over Union between the last seen instance (dashed yellow line) and the current one (green solid line) is greater than zero or the position of the current instance belongs to the same half of the frame of the last seen one, the ID is re-assigned

(2) Check the deleted instances list to assign the same ID of an already seen instance. The correspondence between the two instances is based on the degree of overlap between the two instances: if it proves to be non-null, the identical ID is assigned (Fig. 6). Additionally, instances that belong to the same vertical half of the frame are matched with the same ID.

(3) Create a new instance.Thus, for every frame, a list of instances is generated, each characterized by a consistent ID, that remains coherent across consecutive video frames.

## Experimental settings

The developed method is tested on 10 different videos of length 8 s (approximately 250 frames) anastomosis procedures, balanced between in vivo and in vitro conditions. Furthermore, three different detection backbones, in addition to the proposed one, are used to validate the efficacy of the developed data association module. Specifically, these detection networks are (1) the FastRCNN [32], which is a state-of-the-art object detection model that leverages a ResNet50 backbone and an efficient feature pyramid structure (FPN) to detect and classify objects in image at different scales; (2) YOLOv5 [33]; and (3) YOLO-NAS [27], which are the state-of-the-art one-stage detectors. All these models have pretrained weights and they were fine-tuned on a private balanced dataset of microsurgery composed of over 2000 images, with resolution 1920x1080, in which the Symani®surgical instruments were manually annotated with non-oriented bounding boxes. These images were divided such that 70% of the data are used in training and fine-tuning phase of the network, 20% for the validation and the 10% for testing. Additionally, we augmented the dataset through (1) geometrical transformation techniques and (2) color property changes, as brightness and contrast adjustments and Gaussian blurring. All the networks were trained and tested on a Linux server with an Nvidia A100 GPU based on the Ampere architecture [34].

The metrics used to validate our approach are listed in the following. As first metric, we compute the inference speed *t* as the sum of time for detection ($t_d$) and time for data association ($t_a$).

3 $$\begin{aligned} t = t_d + t_a \end{aligned}$$

The multiple object tracking accuracy (MOTA) is the most widely used metric since it combines three sources of errors and it is defined as:

4 $$\begin{aligned} \textrm{MOTA} = 1 - \frac{\sum \limits _{t=0}^{N}\textrm{FN}_t + \textrm{FP}_t + \textrm{IDSW}_t}{\sum \limits _{t=0}^{N}GT_t} \end{aligned}$$

where *t* is the frame index, *N* is the number of frames, *FN* is the number of false negatives (i.e., missing detection), *FP* is the number of false positives (i.e., wrong detection), *IDSW* is the switch error and *GT* is the number of ground truth instances.

The multiple object tracking precision (MOTP) is the average dissimilarity between all true positives and their corresponding ground truth targets. For bounding boxes overlap, this is computed as:

5 $$\begin{aligned} \textrm{MOTP} = \frac{\sum \limits _{t=0}^{N}(\sum \limits _{i=0}^{S}\textrm{IoU}_{t,i})}{N} \end{aligned}$$

where *i* is the instance index, *S* is the total number of instances in a specific frame and *IoU* is the Intersection over Union between matching bounding boxes and it is computed as the area of intersection divided by the area of union.These last two metrics [35] were also evaluated separately for in vivo and in vitro/mock videos to get different behaviors between these two conditions.

Since all the videos are recorded with constant zoom level, the increment (or decreasing) of the instances’ area in consecutive frames should be stable and comparable. Even though this assumption is valid in most frames, the tracking algorithm is not fooled when the instrument moves toward the edges of the image, where inevitable changes occur as the instrument goes out of focus.

Thus, a new metric called mean of the sum of consecutive area variation (MSCAV) is computed to measure the area variation of each detection in consecutive tracking frames and to evaluate the effect of introducing a weighted sum of instances. Concretely, we calculate the area of each identified instance, subtract the area of the previously matched one, and normalize this difference by the latter’s area to obtain a percentage of increase or decrease. In formula:

6 $$\begin{aligned} \text {MSCAV} = \frac{\sum \limits _{t=1}^{N}\left( \sum \limits _{i=0}^{S}\frac{\textrm{Area}_{i,t} - \textrm{Area}_{i,t-1}}{\textrm{Area}_{i,t-1}}\right) }{N} \end{aligned}$$

where Area is the product between width and height of the given instance. A substantial distance from zero indicates a significant variation in the area, leading to excessive oscillation of the instances in the video.

## Experimental results

**Fig. 7:**
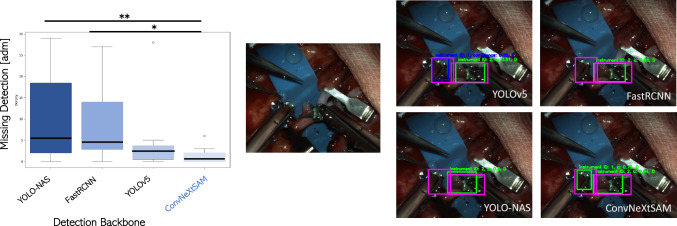
On the left, the distribution of missing detection in the test videos is reported. On the right, an example of the ability of the proposed method to get the small details in the image respect to the state-of-the-art approaches. The green bounding boxes represent the prediction, the magenta ones represent the ground truth, and the blue one is the prediction provided by the Kalman filter

To assess the efficacy of the proposed data association module, different degrees of accuracy within the detection module, which are quantified by considering the frequency of predictions made by the data association module, were examined. Evidently, the proposed method exhibits the minimal incidence of missing detection ($1.7\pm 1.71$ instances per video), showcasing a notable reduction when compared to state-of-the-art approaches (Fig. 7, left). A t test on the results shows a significantly difference of the proposed detector with respect to YOLO-NAS and FastRCNN.

A case of missing detection is reported in the right side of Fig. 7 where it is appreciable the capability of the proposed method to focus on small details in the input image: the only network able to capture the left arm is the proposed one.

**Fig. 8:**
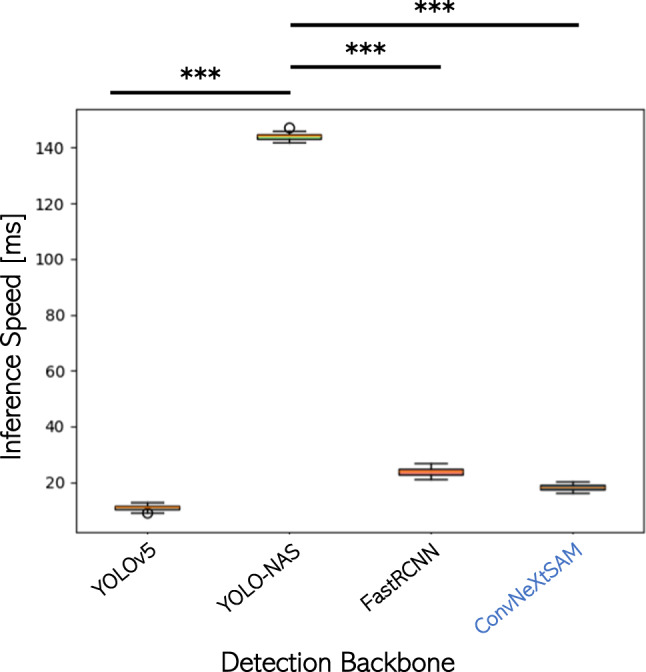
Inference speed: as the complexity of the model increased, also the speed, which is the sum of detection and association time, increases. However, our method shows a real-time behavior

The inference speed of the different approaches that is composed of detection time and data association time shows a result that resemble the complexity of the different networks, i.e., YOLO-NAS is the slowest ($144\pm 0.18$ ms), while YOLOv5 is the fastest ($11.1\pm 0.01$ ms). However, our approach shows an inference speed of $18.26\pm 0.96$ ms and, thus, we can assert that our method is suitable for real-time tracking since the frame rate of the used optical microscope is 30 frames/s (Fig. 8).

The ANOVA statistical test shows a p-value lower than 0.05 (*p*-value=$1.8e^{-87}$) meaning that a significative difference between the methods can be found. The statistical *t* test confirms that the result of YOLO-NAS is significantly different with respect to the other methods that, thus, can be considered as interchangeable.

**Fig. 9:**
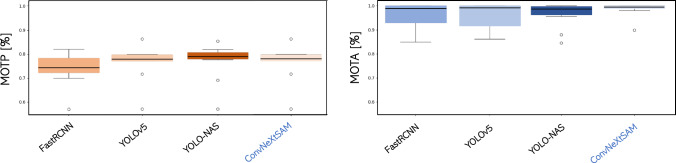
Boxplots of the evaluation metrics: for each detection backbone tested, the multiple object tracking accuracy and the multiple object tracking precision are reported. The former evaluate the number of missing objects and false values respect to the total number of ground truth objects, while the latter evaluate the mean value of the overlap between ground truth bounding boxes and predicted ones

Figure 9 reports the value of the MOTP and MOTA metrics, mediated on all the test videos, for the proposed approach respect to the state-of-the-art methods. The MOTP mean values and MOTA mean values over the 10 videos are ($0.74\pm 0.09\%$ and $0.96\pm 0.05\%$), ($0.73\pm 0.06\%$ and $0.96\pm 0.06\%$), ($0.73\pm 0.05\%$ and $0.96\pm 0.06\%$) and ($0.74\pm 0.06\%$ and $0.99\pm 0.03\%$), respectively, for FastRCNN, YOLOv5, YOLO-NAS and the proposed method. Thus, all the tested methods show similar mean values, proving the robustness of the developed data association module.

Indeed, from the ANOVA statistical test, we obtain no statistical difference between these results, meaning that the data association module works properly, solving the weakness of the detection phase. However, from the boxplots in Fig. 9 it is clear that the variance of the proposed approach is significantly lower than the others three methods, as confirmed by the ANOVA statistical test and the t tests. Thus, our approach is much more repeatable and suitable for different scenarios.

**Table 1:** In vitro samples: results of the metrics for the different tested methods

Metrics	In vitro samples results			
	MOTP [%]	MOTA [%]	MSCAV [%]	Time [ms]
FastRCNN	$0.75\pm 0.02$	$0.98\pm 0.03$	$3.10\pm 1.29$	$23.91\pm 0.08$
YOLOv5	$0.75\pm 0.07$	$0.96\pm 0.06$	$1.71\pm 0.51$	$11.10\pm 0.03$
YOLO-NAS	$0.75\pm 0.07$	$0.92\pm 0.08$	$2.25\pm 0.93$	$144.13\pm 0.49$
ConvNeXtSAM	$0.78\pm 0.04$	$0.99\pm 0.01$	$1.65\pm 0.79$	$18.30\pm 0.12$

Table 1 reports the value of the considered metrics on the mock videos, while Table 2 is focused only on videos taken on in vivo conditions.

**Table 2:** In vivo samples: results of the metrics for the different tested methods

Metrics	In vivo samples results			
	MOTP [%]	MOTA [%]	MSCAV [%]	Time [ms]
FastRCNN	$0.73\pm 0.08$	$0.95\pm 0.06$	$2.79\pm 2.93$	$23.95\pm 0.18$
YOLOv5	$0.73\pm 0.06$	$0.96\pm 0.06$	$1.62\pm 0.40$	$11.10\pm 0.04$
YOLO-NAS	$0.72\pm 0.10$	$0.98\pm 0.02$	$2.04\pm 1.97$	$143.93\pm 0.37$
ConvNeXtSAM	$0.72\pm 0.09$	$0.98\pm 0.04$	$1.55\pm 0.56$	$18.20\pm 0.10$

**Fig. 10:**
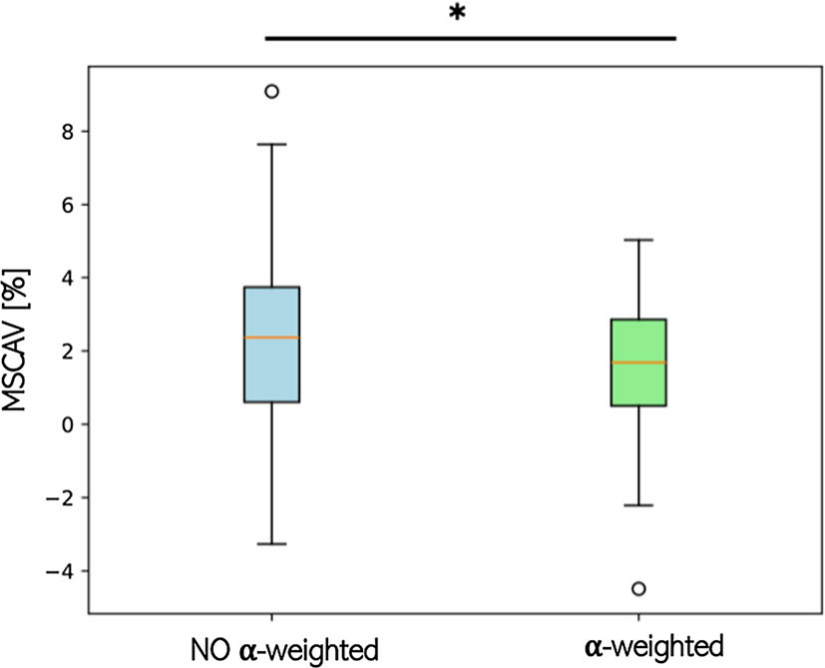
Mean of the sum of consecutive area variation (MSCAV) averaged in the 10 videos with the proposed approach. The introduction $\alpha$ stabilization reduces both the mean value and the variance of the considered metric

The results of the MSCAV metric (see Fig. 10), applied on all the test videos, show a reduction between consecutive frames of 2.04% ± 2.35%, using the proposed approach. Furthermore, the comparison without utilizing the $\alpha$ parameter shows a reduction of 17.13% ± 2.14% of the MSCAV meaning that the oscillation of the instances is decreased between consecutive frames. This results in a more stable transition of the instances along the video.

The ANOVA statistical test shows a p-value lower than 0.05 (*p*-value=0.003) meaning that a significative difference between the usage or not of the $\alpha$ stabilization can be found. The same behavior is also obtained when exploiting the other three detection backbones.

**Fig. 11:**
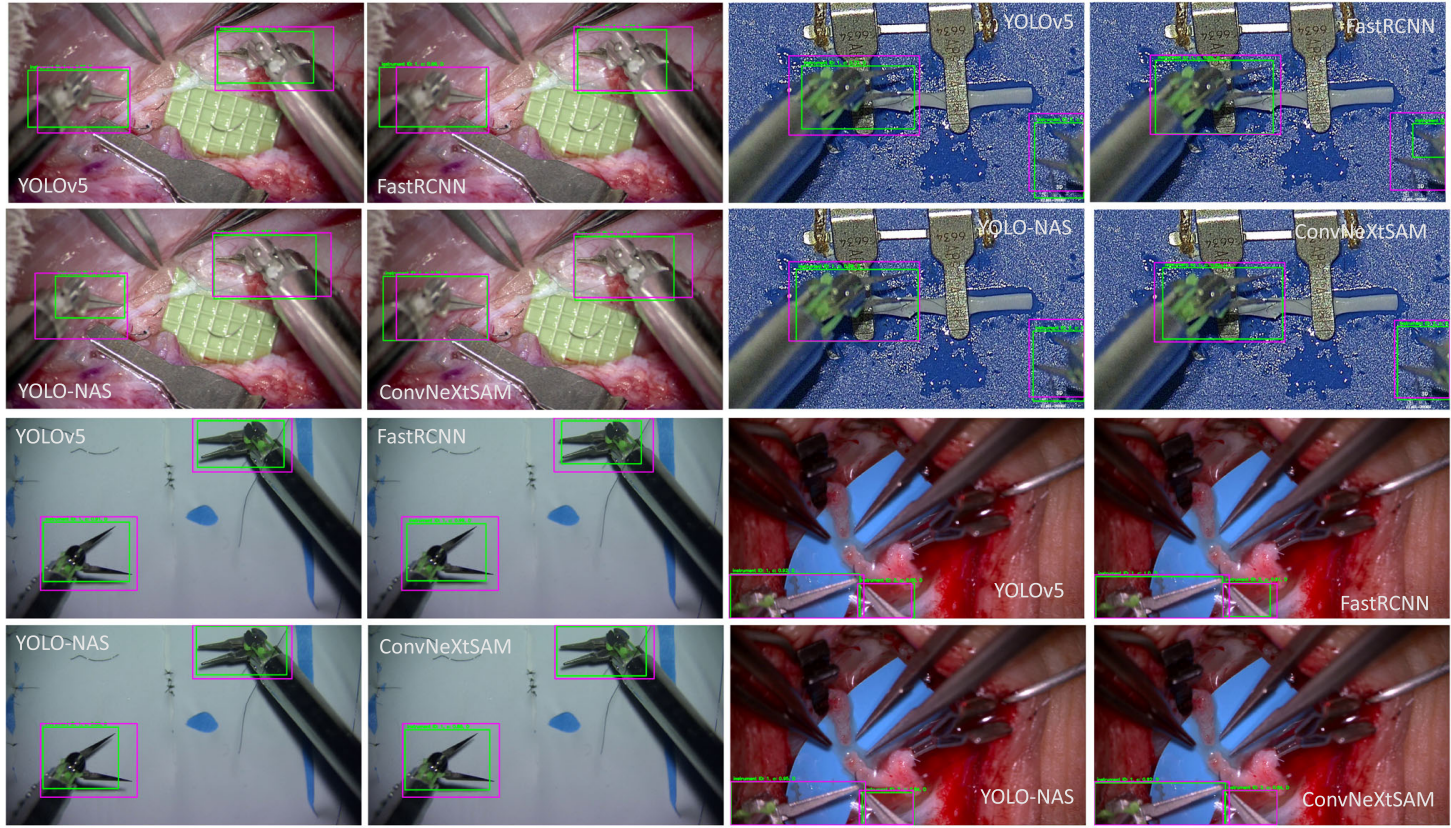
Detection results from four frames taken from the test videos using the four detection approaches. The predicted bounding boxes are reported in green, the ground truth one in magenta

Visual results are reported in Fig. 11, where it is possible to appreciate the ground truth bounding boxes (magenta) compared to the predicted boxes (green) with all the tested approaches in different scenario. It is clear the capability of each method to produce bounding boxes that are more suitable for the size of the instrument’s tips, with respect to the ground truth ones, whose area is found to be broader.

## Discussion and conclusion

In this paper, we proposed a dual-instrument tracking algorithm based on an innovative detection module and a robust data association module for microsurgery robotic instruments. From the test performed, our detection system demonstrates the ability to diminish the operational demands on the Kalman filter, resulting in reduction in the computational time and in the number of missing detection. This means that the developed method exhibits the capability to enhance detection results in comparison to state-of-the-art algorithms while concurrently sustaining real-time performance, despite the fact that the detection network exhibits a higher level of complexity in comparison to YOLOv5 and FastRCNN.

To explain the result of the MOTP obtained through our approach, the methodology employed in generating ground truth annotations should be considered. Truly, a margin between the edges of the instances and the ground truth bounding boxes is introduced, while, as visible in Fig. 11, the prediction of the proposed method perfectly matches with the shape of the instrument. Also, the presence of occluded and not clearly defined instrument (Fig. 11, bottom right) can lead to a decreasing of the predicted area respect to the ground truth. However, the tracking of the two robotic arms remains feasible since they are correctly detected. In general, an overall improvement in detection compared to the current state-of-the-art is evident from the images presented in Fig. 11, both in vivo and in vitro conditions, and in Fig. 7.

Furthermore, Tables 1 and 2 show comparable performances in the two tested scenarios, and this guarantees a general applicability of the proposed algorithm. Finally, the developed methodology effectively stabilizes the area of the instances between consecutive frames, as confirmed by the MSCAV metric, thereby facilitating a considerably smoother transition.

The first limitation of this study relies on the usage of the detection module in each frame, leading to a considerable computational burden. A possible solution can be to invoke the detection module with a lower rate in dependence of the kind of environment and employ the Kalman filter to predict in the intermediate frames. The second limit of the work is its applicability only in a condition of a maximum two robotic arms, while excluding the tracking of manual instruments entering the surgical field. Considering also a third class of manual instrument of assistance proves to be advantageous in notifying the surgeon of both the existence of these instruments and the potential occurrence of occlusion.

Future work will involve the integration of closed-loop control mechanisms to dynamically adjust the camera’s positioning in response to the information generated by the tracker module. In this context, a visual-servoing image-based pipeline will be implemented to ensure that the optical center of the camera aligns with the surgical instruments, placing them at the center of the scene.
